# RecMin Variograms: Visualisation and Three-Dimensional Calculation of Variograms in Block Modelling Applications in Geology and Mining

**DOI:** 10.3390/ijerph191912454

**Published:** 2022-09-29

**Authors:** Arturo Buelga Díaz, César Castañón Fernández, Gonzalo Ares, Daniel Arias Prieto, Isidro Diego Álvarez

**Affiliations:** 1 Mining Exploitation and Prospecting Department, University of Oviedo, 33004 Oviedo, Spain; 2 School of Mining, Energy and Materials Engineering, University of Oviedo, 33004 Oviedo, Spain; 3 Department of Geology, University of Oviedo, 33005 Oviedo, Spain

**Keywords:** mining, variogram map, geostatistics, kriging, mineral deposit

## Abstract

Variogram calculation is a fundamental tool for studying ore grade data in mineral deposits. It allows the discovery of hidden structures within the data and preferential directions of mineralization where the geological continuity is longer. The variogram allows us to classify samples and to define both the search radii for interpolation and the use of kriging as an interpolation and resource classification method. It is not difficult to use it in one dimension; complexity increases in two dimensions as the necessity to search for possible grade anisotropies in all directions arises. Three-dimensionally it is even more difficult to try to define the parameters of lag, bandwidth, and tolerances that define the data of the variogram calculation algorithm. There are rules of thumb to help in the development, but a trial-and-error approach is used in order to find enough sample pairs to allow the generation of variograms truly representative of the data. In this paper, two strategies are shown to help in variogram construction, the use of variogram maps and the graphical representation of the pair search areas (cones or pencils). The freeware tool RecMin Variograms has been developed and is freely available for download at its website; it is easy to learn and use. R code based on RGeostats libraries is used to check the operation and results of RecMin Variograms. Applying geostatistics to mineral deposits is essential to know the areas with the highest grades, thus allowing exact planning of future mining exploitation and minimizing mine footprint and environmental impact.

## 1. Introduction

Ore deposits must be studied and quantified prior to their exploitation so that a mining feasibility project can be developed to guess if the deposit can be profitable.

Based on previous information (geophysical prospecting, surface sampling, trial pit, vertical, inclined, or directional drilling), an attempt will be made to establish a deposit model, firstly creating a lithological model that defines the lithology at each point of the mining domain, and that can be shown through geological maps and sections.

The mineralized bodies, and the geological structures that surround them, will be crossed by drill holes and inclined perforations that allow the intersected material (the sample cores) to be recovered in an intact form.

The cores are analyzed to find their lithology, density, geotechnical characteristics, and ore content (this is, the ore grade), creating interval tables which are then homogenized appropriately to achieve quasi evenly spaced samples, a process called compositing. The values marked by the composites in the three-dimensional space will generate a cloud of points as a three-dimensional scalar field.

**Figure 1:**
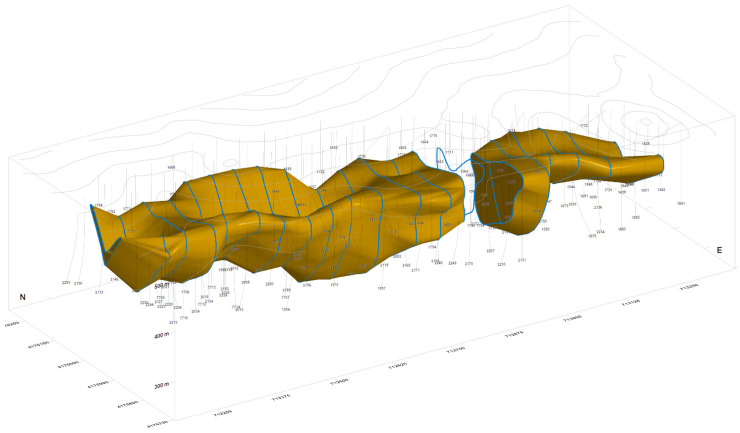
Topography, boreholes, and 3D model of “Salomon” ore body in the Riotinto deposit using RecMin software. Notice the massive size. Coordinates are expressed in meters.

Once the topographic surface is defined, the modelization of the underneath geological structures has the aim to define the continuity of the structures from the composite data. It will be a process of “connecting the dots” between sections marked by polylines surrounding zones of equal lithology or above a grade value, and then connecting the polylines with triangle meshes, then defining ore bodies, veins, or mineralization haloes. This process will be done manually (Figure 1, RecMin software) or semi-automatically with the help of implicit modeling algorithms [1] (commercially available in applications such as Leapfrog, Minesight Geologic, and Surpac Dynamic Shells). The result is a three-dimensional solid, a closed surface formed by a triangle mesh.

Once the shape of the mineral bodies is known, the modeling process objective is to know at each point within these bodies the grade of the profitable element, this is, the concentration of the element per unit mass of the ore. With these data, the deposit value can be estimated, determining the tonnage of the resources and their average grade. Needless to say, the actual grade is only known at those points of the solid through which a borehole passes and their core samples have been analyzed.

In the so-called “block model” methods, the ore bodies inside are discretized using parallelepipeds. The vertexes, the edges, and the midpoints or the centers of these parallelepipeds will be used as grade estimation points from the known data in the composite point cloud through an interpolation procedure.

**Figure 2:**
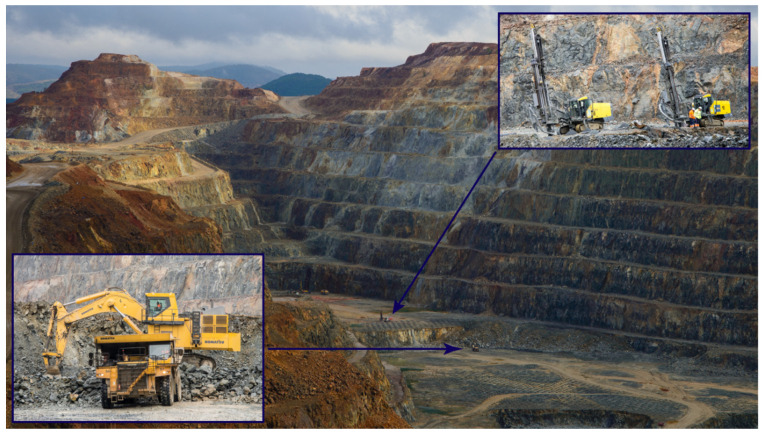
Cerro Colorado pit, Riotinto pollimetalic mine. The mineralized body shown in the previous figure lies approximately in this area.

In a second phase, an economic model will be made, which will allow us to know, at each coordinate, the presence of an element of economic interest (“metal” from now on), its associated grade, and the feasibility of its economic extraction. There could be high-grade resources but at such a depth that it will not be profitable to extract them by open-pit mining methods (method shown in Figure 2). There could be shallow resources, easy to extract, but of such low grade that it will not be profitable to mine them. In the case of open-pit mining, the most profitable pit will be the so-called ultimate pit, that is, the biggest pit that will allow resources to be extracted, maximizing profit, and its volume will define the mining reserves. The process of calculating the ultimate pit, this is, translating resources to reserves, is called optimization.

Block models have been used to calculate resources and reserves for at least 40 years; although there was a limitation regarding the number of blocks in terms of the size of the generated databases and the processing time to use them, these limitations have been surpassed given the speed of current computers and the easiness of managing large databases and associating them with fast three-dimensional drawing systems.

But the main calculation that underlies all these modelization and optimization processes is the interpolation of the composite data to calculate each block value. Interpolation parameters, such as the maximum and minimum number of samples used, the search radii at each of the main cartesian directions, or the interpolation method, must be defined, and this selection will have a strong influence on the result of the calculation. It is also advisable to use a method to estimate somehow the error of the interpolation that will classify the quality of the estimation process.

This is not a simple or obvious process and usually involves the branch of science known as geostatistics, trying to determine the grade change through the mining domain using variogram graphs. This paper presents a new software tool, “RecMin Variograms,” that allows the visual study of the three-dimensional variogram, actually comparing the position of the composites to the shape, length, and orientation of the search bins that are used to calculate the variogram. The results of the variography study are used to perform the interpolation, no matter if it is by simple nearest neighbor methods (where geostatistics just helps in the search radius definition) or complex stochastic methods such as conditional simulation [2].

Applying geostatistics to mineral deposits is essential to know the areas with the highest grades, thus allowing exact planning of future mining exploitation that will have a direct effect on the environmental impact of the mine. Some of the objectives when reducing these environmental impacts are to reduce the footprint or area of mining, reduce waste and, therefore, the size of the tailings dams or adjust the most effective metallurgical techniques in the treatment phase. Metallurgy is one of the stages with the greatest environmental impact.

## 2. Interpolation and Variograms

The study of the dissemination phenomena with spatial correlation started at the beginning of 1950 with the works of Sichel [3] (studying the gold content in south African mines) and Krige [4] (proposing a moving average interpolation technique to be applied in those gold deposits). The rigorous formulation and the solution to the problem was given by Matheron [5,6] and applied to the development of oil and gas reservoirs in the Ecole des Mines de Paris. Seems like the development of geostatistics is firmly linked to mining activities, and these methods are now used in many branches of technology and science, from fisheries resource estimation to image processing [7]. 

We will assume the reader is aware of the main interpolation techniques used in deposit modeling. We firmly believe that a quick geostatistics analysis, at least an omnidirectional variogram, is always necessary as it helps to define the interpolation radius, no matter the interpolation method used. The reality is that the mining sector does not use it frequently, as it is not mandatory to perform the widely used IDW (inverse distance weighting) or NN (nearest neighbor) interpolation methods, which seems to be the most frequent method used, as Maxwell points out [8].

On top of that, the classification of the resources, this is, the estimation of the quality of the interpolation, is not always performed or clearly specified. According to Silva and Boisvert [9], less than 42% of the mineral resources studies issued in 2012 in Canada had enough information to determine the technique used for classification or any classification method at all. Of this 42%, only 2% used the kriging variance (a method that forces a prior geostatistic analysis), while 40% used geometrical methods such as search neighborhood or drill-hole spacing.

**Figure 3:**
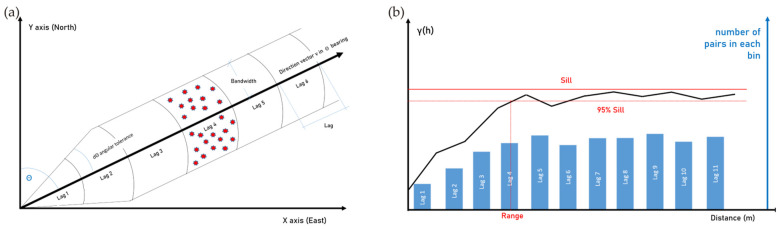
(**a**) Typical variogram that shows sill and range. (**b**) Division of sample pairs into bins for variogram calculation. Adapted from Deutsch and Journel [11].

The geostatistics study begins with the calculation of the variogram graph in selected directions of the three-dimension space. Figure 3 (image left) shows the samples (red dots) included in one of the search bins where the data are classified. The empirical variogram is calculated from the sample data u, as shown by Calder and Cressie [10], and from the equation below, for a vector h that identifies the spatial separation of two samples:

(1) $$2\cdot \gamma_{Y}\left(h\right)=\frac{1}{\left|N\left(h\right)\right|}\cdot \underset{N\left(h\right)}{\sum }{\left[Y\left(u_{i}\right)-Y\left(u_{j}\right)\right]}^{2}$$

where *N*(*h*) is a subset of the samples that satisfy that they are separated by a vector h.

(2) $$N\left(h\right)=\left\{\left(u_{i},u_{j}\right):u_{i}-u_{j}=h\right\}$$

And where |N(h)| denotes the number of unique pairs of locations in N(h) and $\gamma_{Y}\left(h\right)$ is the variogram of the function $Y\left(u_{i}\right)$.

In the case of samples located over non-regular meshes, the number of pairs located exactly at a distance defined by the vector h (given in a certain three-dimensional direction defined by its azimuth and dip, as can be seen in Figure 3a) can be very small and will not give useful results, so the space is divided into zones defined according to this vector h (zones called lags) affected by geometric restrictions:

1. Directional tolerance, dƟ, to one side and the other of the direction Ɵ marking the course.
2. Lag tolerance.
3. Bandwidth.
4. Maximum search distance.

A typical variogram is shown below (Figure 3b). The horizontal axis shows the “lag” or distance between samples considered and defined by the h vector. The vertical axis shows the value of γ(h). A common practice is to show also the number of pairs that are being used in each calculation bin in order to check if enough data have been captured once the geometric restrictions referred to above are applied. The number of pairs is shown in a bluish column graphic on a secondary axis.

The variance value where the variogram curve flattens is called the “sill.” The distance at which the sill is reached is called the range and is a measure of the minimum distance where no relationship is considered to exist between the samples under consideration.

The vector h will have a direction Ɵ which marks the heading. As it will be very difficult for pairs of samples to fall exactly within this line, a tolerance dƟ is set, which can be initially estimated, following Deutch [12], at a value Ɵ/2. When using an angle tolerance of 90°, we obtain the so-called omnidirectional variogram. Reducing the angle tolerance increases the accuracy, and increasing it will improve the stability of the calculation because of the overlapping of the selection zones. Deutch recommends tolerances greater than Ɵ/2 if only a small amount of data is available.

The bandwidth is the maximum distance that sample pairs can be away from the search vector. It is used to limit the number of unreasonable associations and maximize the number of reasonable pairs by creating search cylinders rather than cones. We will refer to these search cylinders as “pencils.” If bandwidth is not used, the search volume is a cone.

The pencil or cone search volumes, marked by vector h and the tolerances described above, are divided into sections of length “l” called “steps” or “lags,” again affected by a tolerance dl. Points at a distance greater than or equal to l-dl and less than or equal to l+dl are used to calculate the experimental variogram. If the total length that the vector h runs through searching for samples is called “D,” Journel and Huijbregts recommend [13] that the number of lags, “N,” be such that N-l < D/2. 

According to Deutch [11], the choice of the lag “l” can have effects on the calculation. A too-small lag will lead to an erratic, unstable experimental variogram, while a too-large one will not show the details of the spatial continuity, more specifically, the behavior at the origin of the variogram. An educated guess to select the lag could be the distance between boreholes if drilling has been made on regular grids. The maximum search distance can initially be estimated as half the distance between the furthest samples in the whole domain (Deutch [11]). Although it is not mandatory, the minimum number of pairs at each point in the variogram should, according to Webster and Oliver [14], be 30. If there are fewer than this value, it may indicate that the distance between lags should be increased. 

In which directions should the pairs will be searched for? In mining applications, a plane with greater grade continuity usually appears within the geological model, corresponding to a layer, vein, or seam, and a direction perpendicular to this plane in which the grade continuity, the range, will be much shorter. If no geological information is available, the so-called “variogram maps” shall be used to explore possible continuity directions. Note that in directions parallel to all the boreholes, it will be difficult to intersect pairs, and no results will be obtained.

## 3. Variograms and Variogram Maps in Deposit Modelling

A one-dimensional variogram is simple to develop and interpret. But trying to analyze thousands of samples taken in a three-dimensional domain using variograms involves simultaneously studying multiple vectors h in multiple orientations of space. As explained above, there will typically be a primary plane in which grade continuity has a maximum range value and a plane perpendicular to this (secondary plane) in which continuity will be dependent on that of the principal plane, following Walter’s law [15]. In the case of layered mineralization, the main plane of mineralization will be the seam plane, with high-grade continuity along the layer. In the perpendicular plane (secondary plane), continuity will exist, albeit to a much lesser extent, but with features common to the primary plane, such as small-scale variations (nugget effect) or the shape of the variogram.

The geostatistics study strategy proposed by Gringarten and Deutch [16] and established in the mining industry (e.g., Surpac geostatistics module [17]) is as follows. First, an attempt is made to identify geological structures that may have led to the generation of preferred directions of grade continuity. If this information is not available, two-dimensional variogram maps can be used. By guessing an initial continuity plane, multiple variograms are calculated within that plane, and the covariance or variogram is represented by contours or heat maps. The direction in which the variogram has the largest range, i.e., the direction in which the sill is reached furthest from the origin, will be the primary direction or major axis of the search ellipsoid. The secondary direction, or semimajor axis of the search ellipsoid, will be the perpendicular inscribed in that primary plane to the major axis. This map can be explored in different primary planes until maximum continuity is revealed, although this does not always happen.

If a primary continuity plane has been discovered, the secondary plane of continuity is now calculated, which will be the plane perpendicular to the primary plane and containing the major axis. The direction perpendicular to the primary plane of continuity will be the minor axis of the search ellipsoid.

Therefore, three directions have been defined in the space in which continuity is considered and which marks the orientation of the search ellipsoid. The size of this ellipsoid will be given by the ranges of the respective sills of the three variograms in the three main search directions. The sills and ranges are calculated on the curves of the variogram models, adjusted by means of formulae (exponential, potential, gaussian, or a combination) to the experimental variogram.

The ratio of the range of the variogram in the principal direction to the range of the variogram in its perpendicular direction, contained in the main continuity plane, is called the major/semimajor anisotropy factor. The ratio of the range of the variogram in the main continuity direction vs. the range of the variogram in the direction perpendicular to the main continuity plane is called the major/minor anisotropy factor. This search ellipsoid is the one that defines the variation of the grade in the three-dimensional space that is being studied, and that will allow us to carry out the interpolation.

How is this method effectively used in practice? Is there any commercial or freely available software that saves the always busy geologist or mine engineer the time-consuming programming of these methods? 

There are commercial solutions in the geological and mining field, such as Isatis [18], Surpac [17] or Leapfrog [19], that are free of bugs and are very user-friendly but have high costs. On the other hand, commercial statistical or data processing software (SPSS, SAS [20], Surfer [21]), which, not being specific to geostatistics, could fall short in terms of options, but we cannot say it for sure as we have not used them. There are geostatistics freeware solutions, such as SGeMS [22], which are infrequently updated and released with some known bugs [23]. Finally, R, freeware statistical software par excellence, extendable and configurable ad infinitum, has multiple options [24]. Both SGeMS and R are very powerful and configurable solutions but are complex to learn and manage, and users must have a strong programming profile, which usually confines their use to the academic environment.

R is based on packages, pieces of code that users develop and share as libraries. There are thousands of them from a community of users that is constantly growing [25], probably boosted by the rise of Big Data. We have delved into the R packages that allow the variogram to be calculated and have found several that could suit our needs.

The gstat package by E.J. Pebesma has multiple modeling and visualization tools for geostatistics [26]. The variogram function information shows how the variogram calculation works [27], and it does not suit our needs as it does not allow us to define a search bandwidth. Diggle and Ribeiro’s geoR package [28] allows complex variogram-based geostatistics, but again no bandwidths can be defined [29]. The RGeostats package [30] from MINES ParisTech uses the vario.calc instruction, including the cylrad parameter, where pairs of samples are removed from a bin if they are more than one value apart in the plane orthogonal to the direction of the variogram calculation; that is, it allows the definition of a bandwidth.

## 4. RecMin Variograms

This paper presents RecMin Variograms [31], a free tool for the calculation and adjustment of variograms geared towards geological and mining applications. Throughout this paper, its use will be validated using RGeostats packages.

**Figure 4:**
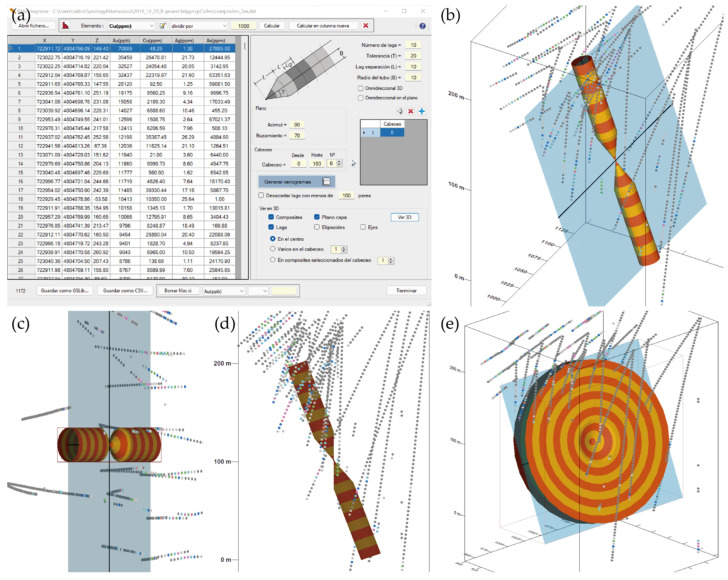
Main screens of RecMin Variograms free software. (**a**) Variography input parameters screen. (**b**) 3D visualization of search parameters. (**c**) Plan view of search parameters. (**d**) Elevation view of search parameters. (**e**) Omnidirectional variogram search parameters.

Based on all the information presented above, it was decided to complete the RecMin geological and mining software with a three-dimensional geostatistics module that would allow the development of projects: RecMin Variograms. After importing data from text files, data filtering can be performed to eliminate outliers, and the frequency histogram can be displayed. On the left side of the following figure (Figure 4a), we can see the frontend where the number of lags and their size, the angular tolerance, and the radius of the search tube (bandwidth) are entered. On the right-hand side of the figure (Figure 4b), we can see the three-dimensional graphical representation of the samples (or composites) together with a colored tube according to the size and tolerance of the lags, which helps in the search for the variographic interpretation and in the choice of the parameters of the search vector and the separation of the lags. We believe that this sort of representation is innovative in the field of statistical software applied to the geostatistics of mineral deposits.

In this case, layered mineralization with an azimuth of 90° and a dip of 70° is graphically observed. Using 10 lags at a 10 m distance, with an angular tolerance of 20° and a bandwidth of 10 m, we observe in the plan and elevation view (Figure 4c,d) that the search area follows the mineralization faithfully and it is very likely to find enough sample pairs to obtain a representative variogram.

A check box allows activating the omnidirectional variogram calculation, which is the one that does not fix the orientation of the search vector, but only its length. Another check box activates the omnidirectional variogram in a plane, which allows the search vector to be limited to a plane defined by azimuth and dip of its line of maximum slope. The lower right image (Figure 4e) shows the representation of the omnidirectional variogram in the plane of azimuth 90° and dip 70°.

**Figure 5:**
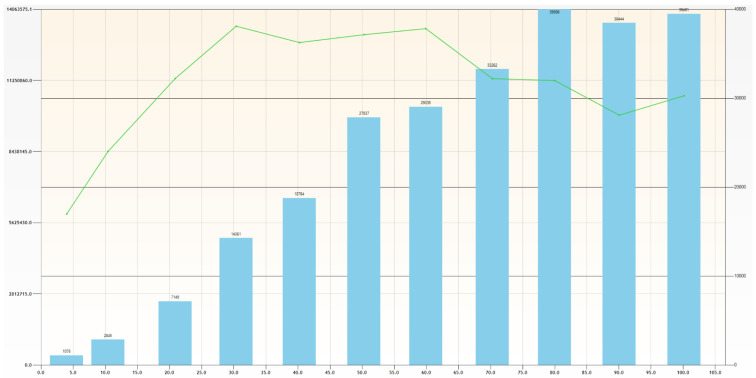
Three-dimensional omnidirectional variogram. The green line is the variogram. The bluish columns are the number of pairs of each calculation bin.

The variogram is calculated and pictured (Figure 5), represented by a green line, with the distance on the *X*-axis and the variogram value on the *Y*-axis. The column diagram shows the number of pairs detected within each lag. We see that the 3D omnidirectional variogram has a sharp rise up to a range of 30 m, with a pronounced sill at 13.5E6.

**Figure 6:**
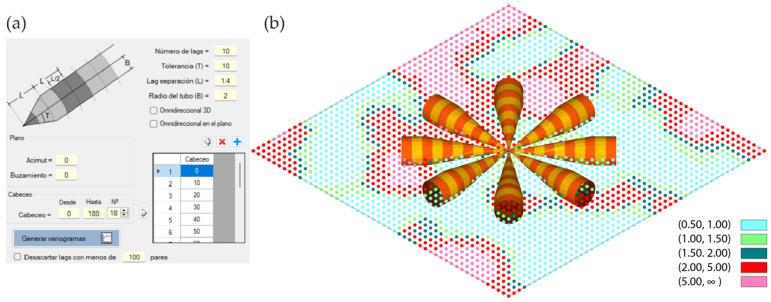
2D example for testing variogram map generation. (**a**). Search parameters. (**b**). 3D drawing showing samples and search pencils.

In order to check whether the software works correctly, a comparison of the results of RecMin Variograms and RGeostats for a two-dimensional example is presented below. Given a Cartesian grid of 2500 evenly spaced data (spacing of 1 m, vertical axis north oriented) with a numerical field varying between 0 and 22.46, that retrieves data from a contaminated soil sampling where Zn percentage has been measured, as shown in the figure below. Contaminated soil sampling is a typical application for 2D variography. After some trial and error, 10 lags with a separation of 1.4 m with an angular tolerance of 10° and a bandwidth of 2 m will be considered for the variogram (Figure 6a). Figure 6b shows both the representation of the pair search pencils and the point data colored as per the grade of the measured data.

**Figure 7:**
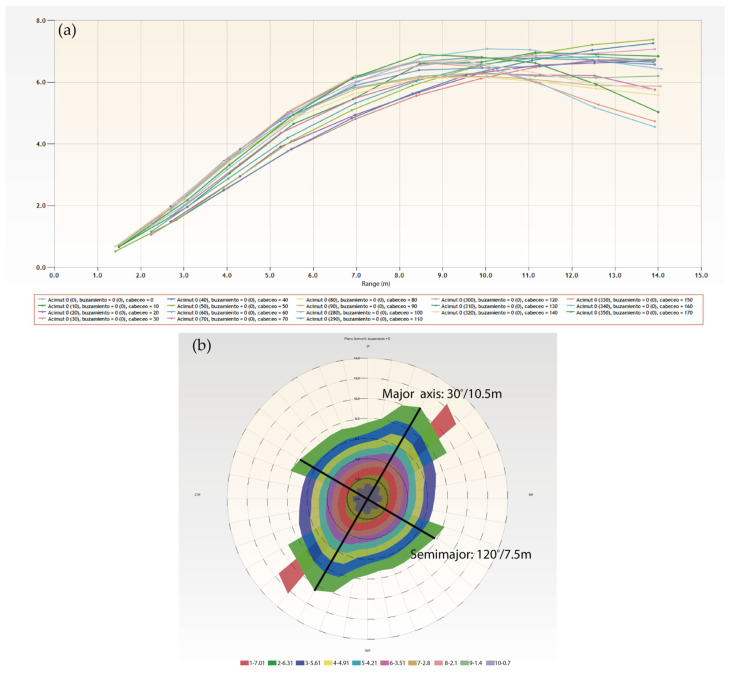
Variogram map calculation in RecMin Variograms. (**a**) Variograms in 18 directions. (**b**) Variogram map.

The calculation is carried out, and the graphical representation of the variogram in RecMin (Figure 7) is obtained. The calculations of 18 superimposed variograms are shown on the left, which implies a jump in pitch angles of 10° since these 18 directions are spread over 180°. All these variograms are shown together in a polar diagram, and their orientation is adjusted as per the bearing of the search pencil, generating the variogram map (Figure 7, right), which shows maximum continuity at bearing 30° (green color, sill value of 6.31) and a range slightly above 10 m.

In this two-dimensional example, the semimajor axis is perpendicular to the major in the calculation plan; following the covariance area value at 6.3, it shows a 120° bearing at a range of 7.5 m.

How to check that the software works correctly? Some of the most used commercial geostatistical solutions as Leapfrog Edge, Surpac, Isatis, or Surfer, allow the calculation of the variogram map, but we intend to use free software here. SGeMS, a free software widely used in mining and petroleum applications, does not have this calculation.

**Figure 8:**
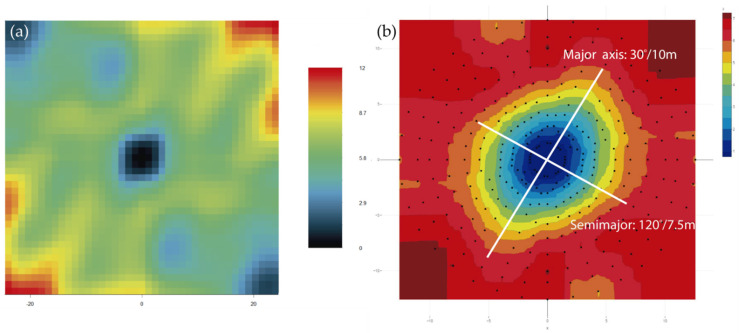
Variogram map using RGeostats instruction vmap.grid (**a**) or using RGeostats automated by authors and drawing via Plotly (**b**).

In R software, the RGeostats library has the vmap.grid function, but it does not allow bandwidth to be defined when segmenting the pairs of data, so we could not use it to compare with our calculation. Nevertheless, we show the result with vmap.grid in the left image (Figure 8a). In order to overcome this RGeostats limitation, we have developed routines that allow iteratively calculating the variograms at each pitch angle. Programming is not complex at all due to the excellent syntax of the RGeostats libraries, but the geometrical translation of the angles is not trivial at all. The direction of the search vector is expressed in Variograms with three angles, bearing, dip and pitch, whereas, in RGeostats, only two angles are used to define the direction. Again, we have programmed it in R code.

The variograms are calculated iteratively for each pitch and stored in a variogram object with the append option. The calculation of the direction of each variogram is not trivial since for each pitch direction within the map plane, direction and dip have to be calculated, and the angles calculated as azimuth give programming problems. This is solved by writing code. The graphical representation in polar coordinates of the stored data of the different variograms is represented in the image on the right (Figure 8b), taking advantage of the contour maps of the Plotly libraries [32].

If we compare the images of the variogram maps generated by RecMin (Figure 7b) and by RGeostats (Figure 8b), we can see that they are very similar. Both variograms clearly show the anisotropy of the grade, with a search ellipse with a principal radius at 30° bearing. On the other hand, this behavior is not seen in the RGeostats default variogram, shown in the previous image (Figure 8a). The semimajor axis is 120°/7.5 m in both RecMin and RGeostats.

## 5. Mining Application in the San Dionisio-Riotinto District

**Figure 9:**
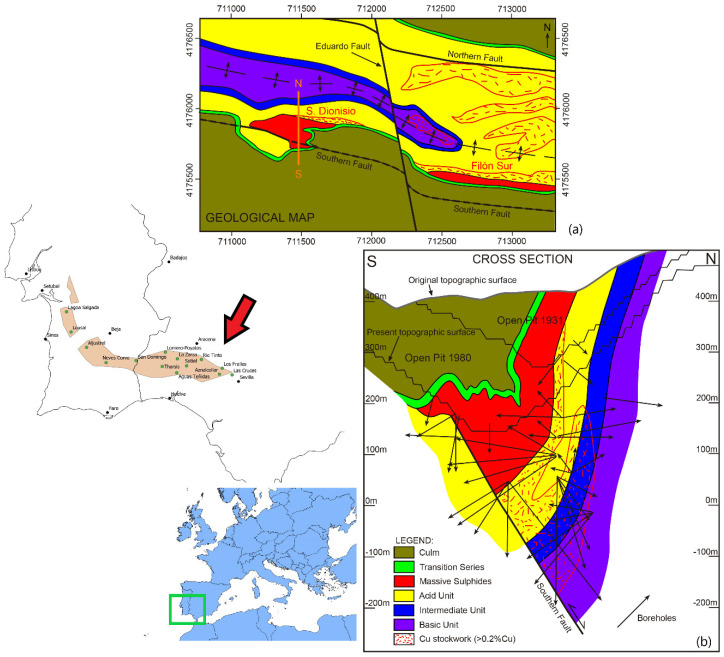
Geological map (**a**) and representative cross-section (**b**) of the San Dionisio lens of the Riotinto Mining District, southwest Spain, within the Iberian Pyrite Belt.

RecMin Variograms will be applied to an actual mineral deposit study case. The data used are from the Riotinto polymetallic deposit [33], San Dionisio area, in the Southwest of Spain (Figure 9). A geostatistical study with RecMin and R{Geostats} will be performed, comparing results. 

The Riotinto Mining District (Spain) is one of the biggest and most massive sulfide accumulations in the world. There are remains of mining activities as old as 1300 BC by the Tartessian civilization [34], long before the roman conquerors arrived in southern Spain and started a large-scale mining facility. The reopening in the XIX century by Riotinto Mining Company Ltd. started modern mining in Spain and lasted from 1873 to 1954. In the last 68 years, the mining district has been in continuous production of pyrite, copper, and gold, with short periods of inactivity. In 2015 the mine was reopened by Atalaya Mining Company to mainly produce copper concentrate.

The Riotinto deposit is formed by seven lenses (mineral bodies) of massive sulfides named San Dionisio, Filon Sur, Filon Norte, Salomon, Argamasilla, Planes, and San Antonio, with a total estimated tonnage of 600 Mt. The rocks in the footwall of the massif sulfides present an intense hydrothermal alteration, with pervasive chloritizacion and silicification in relationship with a pyrite and chalcopyrite stockwork [33].

The Riotinto mineralization is enclosed in a transtensional basin of around 4.5 km long and 0.85 km wide, with direction NNW-SSE, of pre-Variscan age. In this basin was active bimodal volcanism with the development of the Riotinto Volcanic Massive Sulphide deposits. The inversion of the transtensional faults of the early basin was produced during the Variscan orogenesis (Martín-Izard et al. [35]).

The San Dionisio lens is the more occidental in the Riotinto District. It was developed in relationship with the southern fault. The massive sulfide lens was 630 m long with a maximum thickness of 150 m, with a total estimated tonnage of 130 Mt. The actual structure is a syncline generated by the inversion of the Southern Fault what is located to the south of the axial plane (Figure 9).

**Figure 10:**
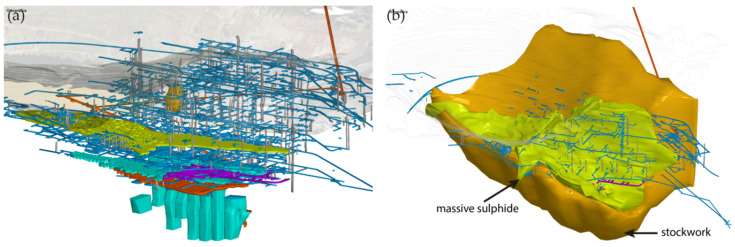
Existing infrastructure in the San Dionisio area (**a**) and superimposed massive sulfide (green) and stockwork (orange) (**b**). The quite remarkable amount of ancient galleries is shown in blue, as well as old stopes.

The San Dionisio area has been mined from 1892 to 1980 by both underground and open pit mining, currently having the Atalaya cut-off as the state in which the open pit mining was left and a significant amount of underground mining infrastructure of ramps, galleries, shafts, and chambers as can be seen in the following images. In the image on the left (Figure 10a), we can see the RecMin Pro modeling of the existing infrastructure to the east of the Atalaya cut, showing the massive amount of ancient galleries in blue. The image on the right shows the modeling of the chlorite solids surrounding the massive sulfides (Figure 10b). Both images show the same location.

**Figure 11:**
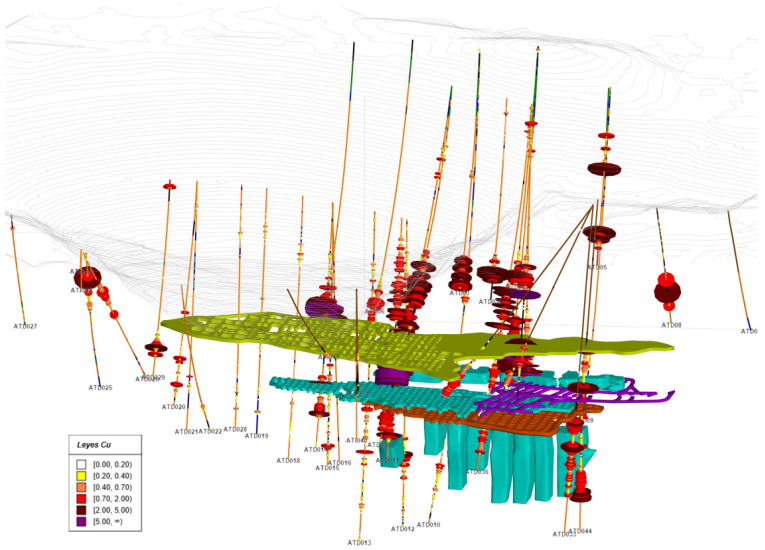
San Dionisio area, Riotinto deposit. New boreholes with information on grades and mined areas.

The information provided by the mining company has been used to develop a geostatistics study: this information includes the boreholes, the updated topography of the area, the geological models of stockwork and massive, and the 3D models of the areas mined by underground methods. Eight hundred eighty-nine old boreholes and 31 new boreholes with analytical data have been used to make the interpolations, of which only the new boreholes have geological information. The image below (Figure 11) shows the current topography (in grey lines, with the historical Atalaya pit on the left), the existing infrastructure of galleries and stopes (greenish and bluish solids), and the boreholes. A greyish color can be seen in the legend below 0.2%, which we consider the cut-off grade, as well as color ranges up to 5.27%, the maximum value. The boreholes are rendered using variable diameter cylinders, proportional to the grade, and also colored per the grade value so that the high-grade zones can be quickly identified in the drawing.

Starting from the 920 boreholes received with analytical information, 5 m composites were generated along the boreholes with the information of the elements Cu, S, Zn, Pb, Ag, As, and Sb in order to carry out the studies and calculations. Since there is no geological information in the old boreholes, the composites have been separated into two types using the Chlorite and Massive models. Of the 13849 composites, 4340 are in massive lithologies and 9509 in stockwork. The geostatistical study will focus on copper, using all composites, without differentiating between massive and stockwork. In our understanding, the common practice in mine geostatistics is to differentiate the studies in different lithologies, that is, to develop different variograms per each lithology. However, in this case, the best continuity results have been obtained without this differentiation.

**Figure 12:**
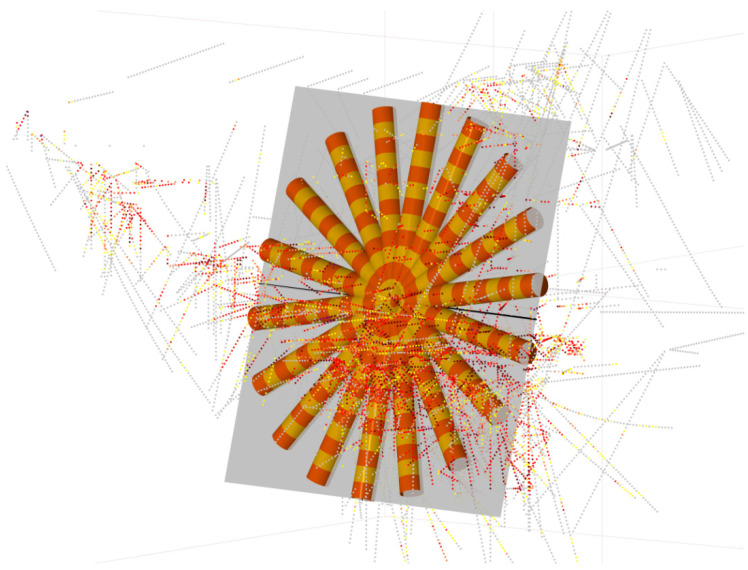
Main plane (azimuth 190°; dip 76°; grey color) and pencils in different pitch directions. Composites are the grade-colored dots.

Studying the geology model and the location of samples, and after adequate inspection of mineralized bodies in the 3D visualizer, it is determined that the plane that best defines the continuity of the grades would be azimuth 190° and dip 76°. Within that plane (Figure 12), several variograms are calculated in different pitches. After several attempts and adjustments, it was found that the best parameters to obtain relevant data were: lag distance: 10 m; number of lags: eight; radius of the search cylinder: 10 m; angular tolerance: 20° and pitch: 20°, that is, 18 search directions in 360°.

**Figure 13:**
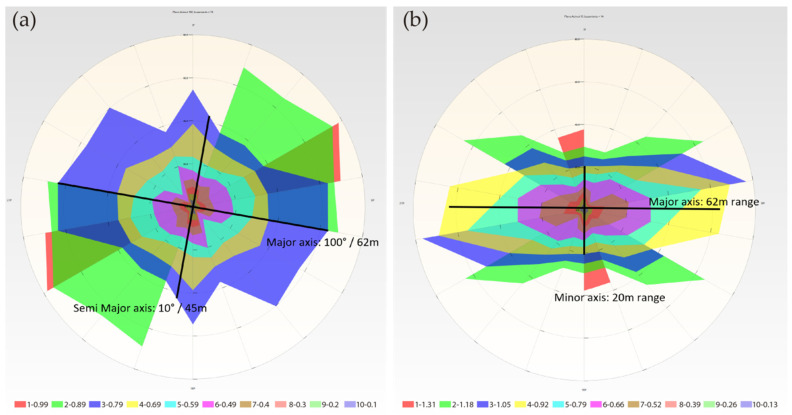
(**a**) Primary 190°/76° (**b**) secondary 10°/14° variogram maps with RecMin Variograms.

The variogram map in this plane would give the following graphic result (Figure 13a). The major axis has a 100° bearing at a range of 62 m, corresponding to a variance value of 0.79. Perpendicular to this direction, the semimajor axis reaches a range of 45 m. Then, the ratio between the major and semimajor search ellipsoid radii is 62/45 = 1.38:1. If we calculate the secondary variogram map in the plane perpendicular to the previous one (azimuth 10° and dip 14°; Figure 13b), it shows an elliptical shape with an approximate 1:3 ratio (62/20 m) between the major and minor axes range.

**Figure 14:**
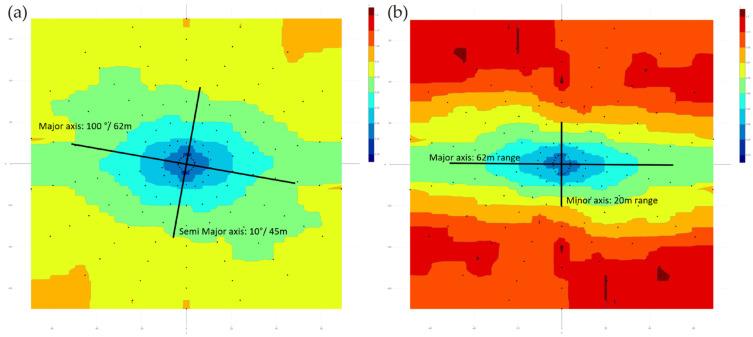
(**a**) Primary variogram maps 190°/76° (**b**) secondary variogram 10°/14° calculated with R{Geostats}.

An equivalent calculation and visualization is obtained in R {RGeostats}, as can be seen below (Figure 14).

**Figure 15:**
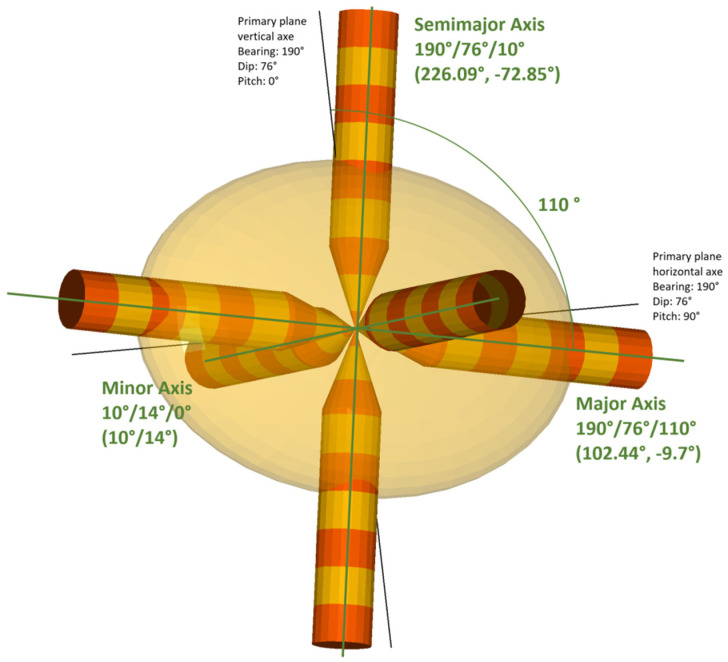
Search pencils in the main directions (the three axes of the ellipsoid) and the calculated search ellipsoid.

Given the continuity provided by the geostatistics study considering all samples, not distinguishing between lithologies, the interpolations will be made without distinguishing between massive and stockwork. The main plane will be the one with a 190° azimuth, a 76° dip, a 100° pitch, and a search ellipsoid with radii 62/45/20 m, or radii ratios of 1 or 1.38:3. This search ellipsoid is shown in Figure 15.

We would like to highlight the importance of angle calculation in a three-dimensional domain, which is much more complicated than in two dimensions. We consider it not trivial or easy to do, and much time has been consumed in checking orientations in both RecMin and R Geostats. The search ellipsoid obtained in the geostatistical analysis has a major axis in 100° pitch inside a plane defined by angles 190°/76°. RecMin variogram angle convention is to measure bearing clockwise from the north direction, to measure dip in positive angles from the horizontal line, and to measure pitch clockwise in positive angles from the primary plane vertical axe, as can be seen in Figure 15. The direction 190/76/100 is equivalent to a 102.44° bearing and a −9.7° dip angle in the more classic notation in three-dimensional space. RecMin Variograms gives information on both angle notations in the results.

## 6. Conclusions

The calculation of the variogram of composites (samples) allows knowing the trends in these grades as a function of spatial orientation, with the main objective of predicting their grade in those areas where it is not known.

The one-dimensional calculation is not geometrically complex, as the variogram is calculated just in one direction. The two-dimensional calculation involves calculating the variogram over the full range of polar angles, from heading 0° to heading 360°. If the scalar field being studied, this is, the grade of an element in the mining domain, has an anisotropic distribution, which is common in mineral deposits due to their seam or vein-shaped geometry, this anisotropy will clearly appear in the variograms, showing sills in the variogram graph at a greater range in the main continuity directions.

In order to generate the variogram, the composites have to be classified in the so-called bins, defined by the maximum search distance, the angular tolerances, and, in the case of using pencils, the search bandwidth. If bins are not used, no relevant variogram will be found as the number of pairs that fulfill a strict range condition between them will not give enough data to calculate the variance value.

The selection of this bin definition parameters is not simple and is guided by the experience of the technician making the calculation, and it usually starts with the adequate study of the geometrical conditions related to the distribution of the composites in space.

In the case of three-dimensional data, it gets even more complicated. It will be a matter of looking for three main directions of anisotropy, which are not always easily recognizable. In this paper, we have shown two strategies that help to determine them.

Firstly, it explains how to elaborate the so-called “variogram maps,” renderings of the variograms in a plane, which will help to determine two of the main continuity directions. In the plane perpendicular to this first one, the variation in the third continuity direction is defined. In this paper, we show the process of creating these variogram maps using freeware software RecMin, firstly with a simple 2D example and finally with a complex actual data set from Riotinto mining district.

Secondly, to help in the definition of the three-dimensional variogram, RecMin Variograms include the three-dimensional representation of the search cones or pencils for the classification of the pairs of samples, which are very helpful in understanding the process of creating and adjusting the variogram. This is a novel method that is not included in the software currently available on the market. It also shows the angles of the main ellipsoid defining radii not only in classical bearing/dip notation but also in bearing/dip/pitch values inside the main continuity plane.

To check the validity of the RecMin Variograms code, calculations are performed both with this freeware and with the RGeostats library of the R software.

The RecMin Variograms software is completely free to download and use and can be found on the RecMin website.
